# Clinical Significance of MRI and Pathological Features of Giant Cell Tumor of Bone Boundary

**DOI:** 10.1111/os.12510

**Published:** 2019-08-18

**Authors:** Liang Chen, Xiao‐lin Shi, Zi‐ming Zhou, Ling‐di Qin, Xiao‐hong Liu, Lei Jiang, Qing‐jiao Zhang, Xiao‐yi Ding

**Affiliations:** ^1^ Department of Radiology Shanghai University of Traditional Chinese Medicine Yueyang Hospital of Integrated Traditional Chinese Medicine and Western Medicine Shanghai China; ^2^ Department of Radiology Ruijin Hospital, Shanghai Jiao Tong University School of Medicine Shanghai China

**Keywords:** Giant cell tumor of bone, Magnetic resonance imaging, Curettage, Immunohistochemistry

## Abstract

**Objective:**

To find new clues to reduce postoperative recurrence after intralesional curettage by studying MRI and pathological features of giant tumor of bone (GCTB) boundaries.

**Methods:**

A retrospective study was performed in the departments of orthopaedic surgery and medical imaging at our hospitals from January 2006 to August 2016. A total of 16 GCTB patients confirmed by pathology were asked to participate in the present study. The age range was from 18 to 44 years (9 women and 7 men). All patients underwent MRI examination. All patients underwent en bloc resection and complete postoperative tumor segments were obtained. Five specimens were obtained randomly at the place of the segments where the GCTB boundary showed different types on MRI. Ordinary HE staining was used for all specimens and we measured the depth of local tumor cell infiltration (240 measurements). Results were expressed as means ± standard deviation. Statistical analyses were carried out with one‐way ANOVA and the Student–Newman–Keuls test. *P* < 0.05 was considered statistically significant. The kappa test was used to analyze the degree of agreement of observers.

**Results:**

A total of 16 patients (median age 30.56 years; range, 18–44 years) with GCTB (the number of distal femurs and proximal tibias was 9 and 7, respectively) were tested. The boundaries of all GCTB cases were composed of clear boundary, relatively clear boundary, and blurred boundary in different proportions on MRI. Based on continuous observation of all MRI, all boundaries were incomplete. The kappa value between two radiologists and two pathologists was 0.91 and 0.88, respectively. The average depth of local tumor cell infiltration in the clear boundary, relatively clear boundary, and blurred boundary groups was 0.42 ± 0.11 mm, 2.85 ± 0.21 mm, and 4.83 ± 0.12 mm, respectively. There was statistical difference among the three groups (*F* = 17.62, *P* < 0.05). There was also statistical difference between each of the two groups (*q*‐value was 8.95, 14.28, and 5.21, respectively, *P* < 0.05). The depth of local tumor cell infiltration with blurred boundaries on MRI was the largest and the depth with clear boundaries was the smallest.

**Conclusion:**

The intralesional curettage boundaries need to be expanded on the basis of different types of boundaries provided by MRI.

## Introduction

Giant tumor of bone (GCTB) is a kind of benign lesion most often found in bone extremities. The biological behavior of GCTB ranges from latent, active to locally aggressive forms and occasionally distant metastasis^1^, ^2^. The biological behavior of GCTB ranges from latent, active to locally aggressive forms and occasionally distant metastasis^1^, ^2^, ^3^, ^4^.

Surgery is the mainstay of treatment for GCTB^1^, ^5^. En bloc resection has been recommended for GCTB. Although complete removal of the lesion provides a low recurrence rate, wide resection requires complex reconstruction of the adjacent joints, which increases the rate of surgical complications and disabilities^6^, ^7^. Intralesional curettage with adjuvants is a feasible first‐choice treatment option for GCTB because of the good function preservation and lower rates of surgical complications^8^, ^9^, ^10^, ^11^, ^12^. Nevertheless, the recurrence rate remains relatively high^5^, ^7^, ^8^, ^9^, ^13^. Previous studies showed that there was no clear relationship between factors such as age, gender, location, and pathologic fracture and recurrence after intralesional curettage. The choice of surgical methods was related to the recurrence rate after surgery^1^, ^14^, ^15^. However, these findings had limited clinical significance in reducing recurrence after intralesional curettage. Some studies demonstrate that the residual tumor cells located in peripheral tissue are important factors of recurrence after intraregional curettage^1^, ^16^. Determining how to define the intralesional curettage boundary and how to clear the residual tumor cells as much as possible have become important research topics.

MRI can not only be used to make a diagnosis of GCTB but also provides a good representation of GCTB boundaries. Detailed and accurate information regarding GCTB boundaries is very important for orthopaedic surgeons. The surgeon needs to know whether the GCTB boundary displayed on the MRI is the intraregional curettage boundary. Thus far, there is limited research in this area.

With improvements in orthopaedic technology and the development of orthopaedic artificial intelligence robots, the definition of the preoperative boundary needs to be more precise so that the residual tumor cells can be reduced as much as possible. All of these issues require a more accurate assessment of the GCTB boundary.

The combination of medical imaging and pathology provides valuable information for the diagnosis and treatment of clinical diseases, which are key tasks for radiologists. Hence, we carried out the present study.

The purposes of the study are: (i) to summarize GCTB boundary features on MRI and pathological sections; (ii) to provide precise information on intralesional curettage boundaries; and (iii) to provide basic data for orthopaedic artificial intelligence surgery in the future.

## Methods

### *Patients and Specimens*

From January 2006 to August 2016, the present study consecutively enrolled 16 patients (9 women and 7 men), with an average age of 34 years (ranging from 18 to 44 years). Their clinical details are summarized in Table 1. All cases were referred by Yueyang Hospital, Shanghai University of Traditional Chinese Medicine, and Ruijin Hospital, Shanghai Jiao Tong University School of Medicine. The inclusion criteria were as follows: (i) patients who were suitable for MRI examination; (ii) all patients underwent en bloc resection and preserved complete pathological specimens; (iii) postoperative pathological findings confirmed as GCTB; (iv) all patients had no other treatment before surgery; and (v) informed consent was obtained from all individual participants who were enrolled in the study. Exclusion criteria were: (i) patients who were unsuitable for MRI examination; (ii) patients in whom surgical treatment was not appropriate; and (iii) the records were incomplete. Based on these criteria, 8 patients were excluded and 16 patients were included.

**Table 1:** Clinical information for all 16 giant tumor of bone cases

Cases	Age (years)	Sex	Localization
1	22	F	Distal femur
2	18	F	Distal femur
3	32	M	Distal femur
4	33	F	Distal femur
5	27	M	Distal femur
6	36	M	Distal femur
7	44	M	Distal femur
8	28	F	Distal femur
9	33	F	Distal femur
10	35	F	Proximal tibia
11	37	M	Proximal tibia
12	22	F	Proximal tibia
13	28	F	Proximal tibia
14	30	M	Proximal tibia
15	34	M	Proximal tibia
16	30	F	Proximal tibia

### *MRI Protocols*

All MRI were obtained using a 3.0T superconducting whole‐body imager (GE Signa Excite system or PHILIPS Ingenia) with a dedicated extremity coil. The following sequences were obtained: spin‐echo T1‐weighted (T1W, TR range/TE range, 450–600/15–20), fast spin‐echo T2‐weighted (T2W, TR range/TE range, 2500–4000/80–120), and fat‐suppressed fast spin‐echo T2‐weighted (T2WI/FS, TR range/TE range, 2500–4000/80–120). The field of view varied from 14 to 18cm. The slice thickness was 5 mm and the interslice gap was 0.5 mm. The number of acquisitions was 2. The imaging matrix ranged from 192 × 256 to 256 × 256. All MRI were analogized in consensus by two radiologists experienced in musculoskeletal MR diagnosis.

### *Surgery Process*

All patients underwent en bloc resection after complete preoperative preparation. After general anesthesia, the lesion was cut from the skin to the tumor tissue layer by layer in the GCTB localization area. After fully exposing the tumor, resection was performed at least 3 cm away from the tumor boundary (naked eye). Structural reconstruction after local was done by autogenous bone (3 cases) or prosthesis replacement (13 cases).

### *Objective Features of MRI*

*Clear boundary*: A clear boundary was defined as a clear, smooth, and continuous low signal line on T1WI, T2WI, and T2WI/FS, respectively.

*Relatively clear boundary*: A relatively clear boundary was characterized by the presence of a low signal line on T1WI, T2WI, and T2WI/FS, respectively. However, the low signal line was not smooth and clear and the low signal line remained continuous.

*The blurred boundary*: A blurred boundary mainly presented as the low signal line being blurred and discontinuous around the tumor on T1WI, T2WI, and T2WI/FS, respectively.

### *Processing of Specimens*

The GCTB segments were cut into segments of 5‐mm thickness according to MRI. Five specimens were obtained randomly at the place where the boundary presented as clear, relatively clear, and blurred on MRI, respectively. HE staining was used for all specimens and we measured the depth of local tumor cell infiltration.

### *Measurement of Infiltration Depth of Local Tumor Cells*

There were three main manifestations on pathological sections.

**Figure 1:**
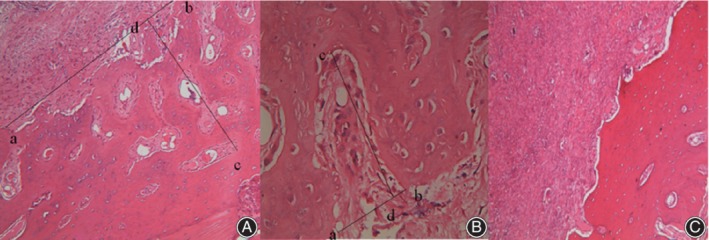
(A) Segment ab was the line connecting the innermost boundary of the tumor and point c was the farthest infiltrating tumor cell in the field of vision. The length of segment cd which was perpendicular to line ab was the farthest infiltrating depth of tumor cells. (B) Segment ab was the connection between two broken ends of the tumor boundary. Point c was the farthest infiltrating tumor cell in the field of vision. The segment cd length which was perpendicular to segment ab was defined as the farthest infiltrating depth. (C) the boundary was clear and continuous. There was no obvious local infiltration of tumor cells into the surrounding normal tissues.

1. The boundary of GCTB was irregular. There were scattered GCTB tumor cells infiltrated in the surrounding normal tissues. In this visual field, point a and point b were the positions of the innermost boundary, respectively. The connection line between the two points was defined as the innermost boundary (segment ab). Point c was the farthest infiltrating tumor cell in the field of vision. The length of the vertical line (segment cd) between the farthest local infiltration cell and segment ab was defined as the depth of tumor cell infiltration (Fig. 1A).
2. The boundary of GCTB was irregular with GCTB tumor cells locally infiltrating into normal tissues. Point a and point b were the two broken ends of the boundary, respectively. The connection line between the two points was defined as the boundary of GCTB. Point c was the farthest infiltrating tumor cell. The length of the vertical line (segment cd) between the farthest local infiltration cell and segment ab was defined as the depth of tumor infiltration (Fig. 1B).
3. The boundary of GCTB tumors was clear and continuous. There was no obvious local infiltration of tumor cells into the surrounding normal tissues. We believed that there was no obvious local tumor cell infiltration (Fig 1C).

### *Observer Study*

All MRI were graded in consensus by two radiologists with 10 years’ experience in musculoskeletal MRI. The endings were described only when both observers could definitively establish a diagnosis on the basis of the images.

Histopathological examinations and measurements were performed by two senior pathologists with more than 10 years of experience in our institution. Each specimen was measured three times and the average value was recorded.

On completion of the retrospective review, the MRI were correlated with histopathological results. The two doctors settled any disagreement through consultation.

## Statistics

Data were analyzed using SPSS (ver. 17.0, Chicago, IL, USA). Results were expressed as means ± standard deviation. Differences in the quantity data among the three groups was tested by one‐way ANOVA test. The comparisons between each of the two groups were tested by Student–Newman–Keuls test. *P* < 0.05 was considered statistically significant. The kappa test was used to analyze the degree of agreement of observers.

## Result

All patients completed MRI examinations and en bloc resection successfully. Pathological characteristics of specimens were typical GCTB pathological features, comprising giant cell tumor of bone stromal cells (GCTSC) with a number of multinucleated giant cells.

**Figure 2:**
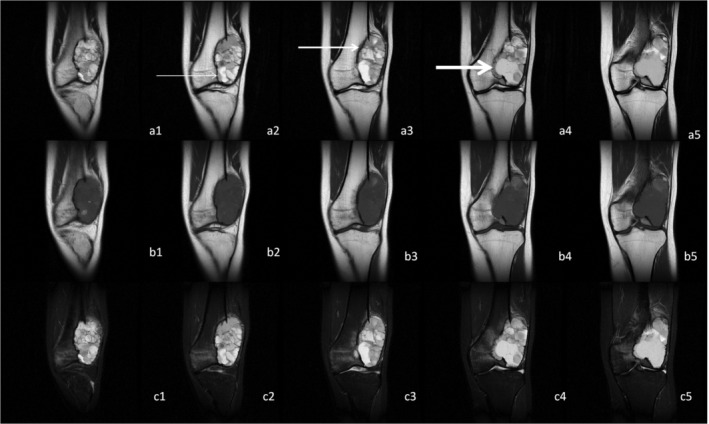
Giant tumor of bone (GCTB) located at distal femur. a1‐a5, b1‐b5, and c1‐c5 were continuous T1WI, T2WI, and T2WI/FS, respectively. The thin arrows, thicker arrows, and thick arrows refer to clear boundary, clearer boundary, and blurred boundary of GCTB, respectively. The boundaries were incomplete with continuous observation of MRI.

**Figure 3:**
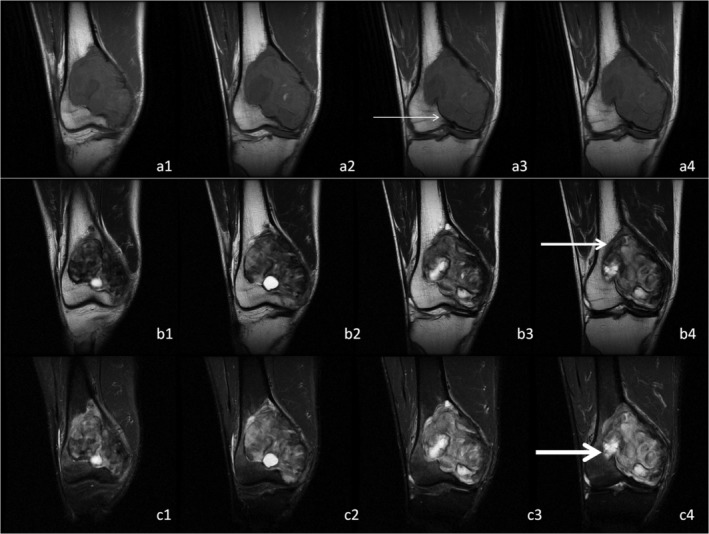
Giant tumor of bone (GCTB) located at distal femur. a1‐a4, b1‐b4, and c1‐c4 were continuous T1WI, T2WI, and T2WI/FS, respectively. The thin arrows, thicker arrows, and thick arrows refer to clear boundary, clearer boundary, and blurred boundary of GCTB, respectively. Outward protrusion of tumor tissue existed in the blurred boundary region.

Two radiologists and two pathologists completed the analysis and measurement of all cases. The kappa value between two radiologists and two pathologists was 0.91 and 0.88, respectively. In our group, the boundaries of all GCTB cases included clear, relatively clear, and blurred boundaries in different proportions. Although some boundaries were displayed as a complete low signal line around the GCTB on some MRI, with continuous observation of all MRI, we found that the boundaries were incomplete (Fig 2 and 3).

### *Clear Boundary*

**Figure 4:**
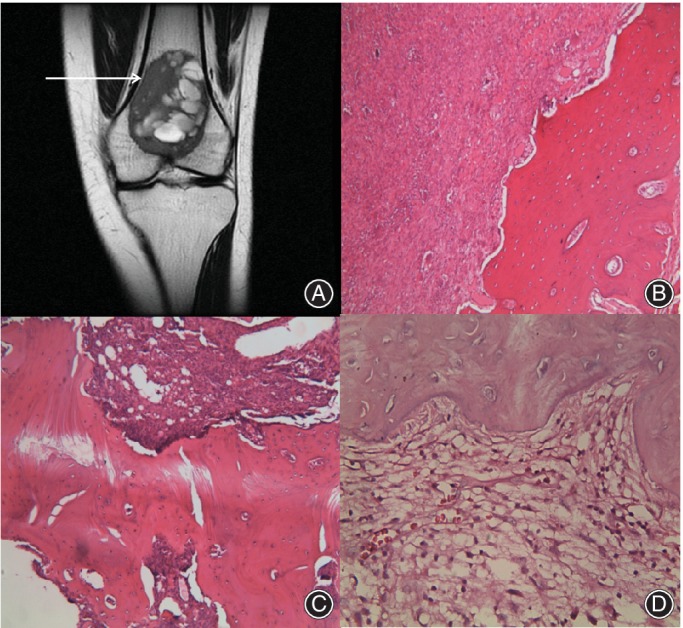
Giant tumor of bone (GCTB) located at distal femora. (A) Arrow refers to the area of samples collection at the clear and smooth boundary. (B) Clear and smooth boundary without local tumor cells infiltration. C and D show clear and smooth boundaries with slight local tumor cell infiltration. (B and C: 100×, D: 400×).

The number of instances of local cell infiltration depth in the range of 0–1 mm and 1–2 mm was 75 (93.75%) and 5 (6.25%), respectively. No case with a depth exceeding 2 mm was found. The average depth of local tumor cell infiltration was 0.42 ± 0.11 mm. This indicated that there was no or only slight tumor cell infiltration with clear boundaries on MRI (Fig. 4).

### *Relatively Clear Boundary*

**Figure 5:**
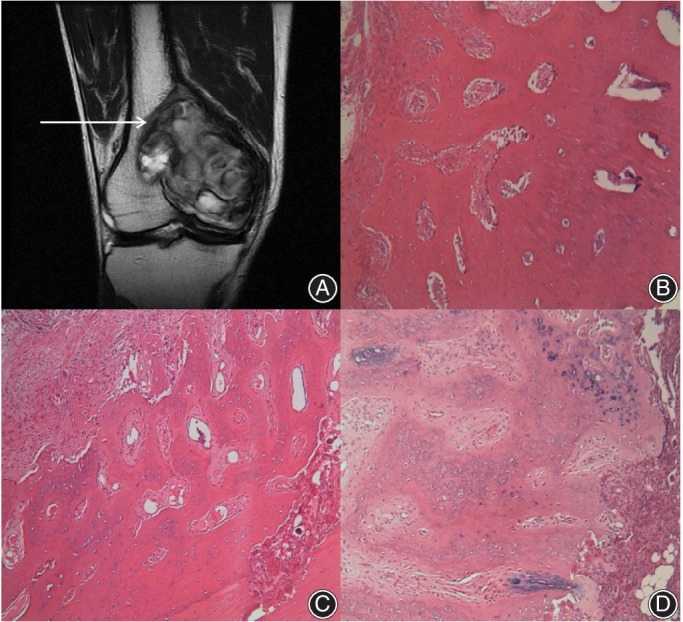
Giant tumor of bone (GCTB) located at distal femora. (A) Arrow refers to the area of samples collection at the relatively clear boundary of GCTB. (B, C, D) showed local tumor cell infiltration. (b, c and d: 100×).

We found that all cases had local tumor cell infiltration. The number of instances of local cell infiltration depth in the range of 1–2 mm, 2–3 mm, and 3–4 mm was 6 (7.50%), 58 (72.50%), and 15 (18.75%), respectively. There was only 1 case where the depth was 4.34 mm. The average depth was 2.85 ± 0.21 mm. This indicated that there was tumor cell infiltration with relatively clear boundaries on MRI (Fig. 5).

### *Blurred Boundary*

**Figure 6:**
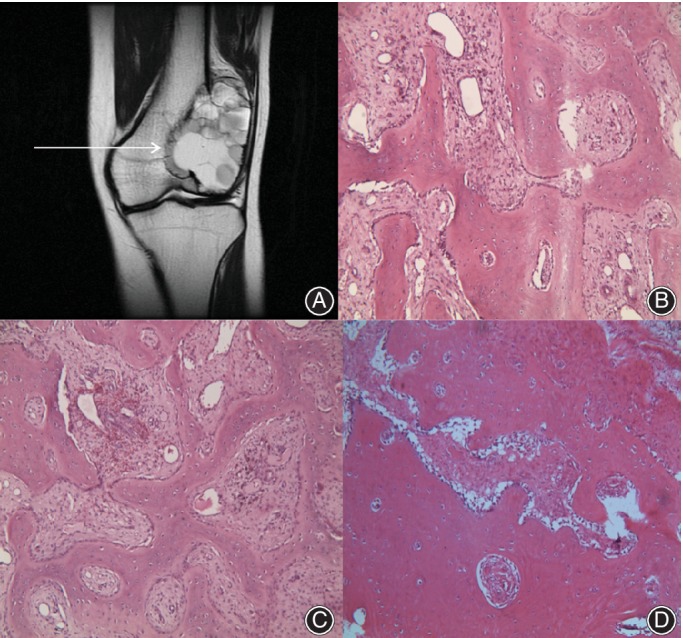
Giant tumor of bone (GCTB) located at distal femora. (A) Arrow refers to the area of samples collection at the blurred boundary. (B, C, D) Obvious local tumor cell infiltration. (B, C and D: 100×).

All cases had local tumor cell infiltration. The number of instances of local cell infiltration depth in the range of 3–4 mm, 4–5 mm, and 5–6 mm was 5 (6.25%), 70 (87.50%), and 5 (6.25%), respectively. There were no cases where local tumor cell infiltration depth exceeding 6 mm or was less than 3 mm. The average depth was 4.83 ± 0.12 mm. This illustrated that there was obvious tumor cell infiltration in the areas with blurred boundaries on MRI (Fig. 6).

Statistical results showed that the average infiltration depth was different among the three groups (one‐way ANOVA, *F* = 17.62, *P* < 0.05). See Table 2. There were also statistical differences between each of the two groups (Student–Newman–Keuls test, *q‐*values were 8.95, 14.28, and 5.21, respectively, *P* < 0.05). See Table 3. The local tumor cell infiltration depth with blurred boundaries on MRI was the largest and the local tumor cell infiltration depth with clear boundaries was the smallest.

**Table 2:** Comparison of local infiltration depth of giant tumor of bone tumor cells with different kinds of boundaries characteristics in MRI (mean ± standard deviation)

Group	Clear boundary in MRI	Relatively clear boundary in MRI	Blurred boundary in MRI
*n*	80	80	80
Depth of tumor cells infiltration (mm)			
0–1	75	0	0
1–2	5	6	0
2–3	0	58	0
3–4	0	15	5
4–5	0	1	70
5–6	0	0	5
>6	0	0	0
mean±SD (mm)	0.42 ± 0.11*	2.85 ± 0.21*	4.83 ± 0.12*
*F‐*value	17.62		
*P*‐value	*P* < 0.05		

* Significant statistical difference (*P* < 0.05).

**Table 3:** Comparison between each two groups

Contrast group	1 and 2*	1 and 3*	2 and 3*
Difference in means	2.43	4.41	1.98
*q*‐value	8.95	14.28	5.21
*P*‐value	*P* < 0.05	*P* < 0.01	*P* < 0.05

1, clear boundary in MRI; 2, relatively clear boundary in MRI; 3, blurred boundary in MRI.

* Significant statistical difference (*P* < 0.05).

## Discussion

Giant tumor of bone is benign lesion most often found in bone extremities. The biological behavior of GCTB ranges from latent, active to locally aggressive forms and occasionally distant metastasis^1^, ^2^. The biological behavior of GCTB ranges from latent, active to locally aggressive forms and occasionally distant metastasis^1^, ^2^, ^3^, ^4^.

MRI can not only be used to make a diagnosis of GCTB but also reveals GCTB boundaries. Our research demonstrated that the performance of GCTB boundaries was inconsistent on MRI. There were three main types of the GCTB boundary demonstrated by MRI: clear, relatively clear, and blurred. Although the boundary of GCTB was displayed as a complete clear low signal line around the tumor on some MRI, we found that the boundaries of all cases were incomplete with continuous observation of all MRI. This is consistent with the biological behavior of GCTB, which is benign and locally invasive. The main reason was that the volume of the tumors was large and the invasion of local tissues had already existed when the patients first visited our hospitals. Few patients with early GCTB present to clinics. At present, the parameter settings of MR scanning sequences are mainly aimed at localization and qualitative diagnosis of GCTB. Based on the current scanning parameters, we could not analyze the details of the boundary accurately just using a low signal thin line because it was small on the MRI. Therefore, we can only make a preliminary classification of GCTB boundary features according to the information provided by MRI. We suggest that the scan parameters need to be adjusted to provide a more accurate representation of GCTB boundaries after completed GCTB diagnosis using regular scan sequences. We carried out adjustment of MR scanning parameters.

Surgery is the mainstay of treatment for GCTB^1^, ^5^. Intralesional curettage with adjuvants is a feasible first‐choice treatment option for GCTB because of the good function preservation and the associated lower rates of surgical complications^8^, ^9^, ^10^, ^11^, ^12^. Nevertheless, the recurrence rate remains relatively high^5^, ^7^, ^8^, ^9^, ^13^. The residual tumor located in peripheral tissue was one of the important factors of tumor recurrence after intraregional curettage surgery^1^, ^16^. Accurate display of GCTB boundaries before intralesional curettage and precise definition of operation boundaries are very important to minimize residual GCTB tumor cells. With the development of artificial intelligence technology, artificial robots will play an increasingly more important role in GCTB intralesional curettage. All this required more accurate data on GCTB boundaries. Doctors require a more detailed and precise definition of GCTB boundaries before intralesional curettage.

MRI can show the boundaries of GCTB clearly. However, is the GCTB boundary displayed on the MRI the intraregional curettage boundary?

The histopathological research showed that the average depth of local tumor cell infiltration in the groups with a clear boundary was 0.42 ± 0.11 mm. This indicated that although GCTB showed clear and smooth boundaries on MRI, some cases already had slight local tumor cell infiltration. The clear and smooth low signal lines displayed on MRI could not fully confirm that there was no tumor cell infiltration. The average depth of local tumor cell infiltration with a relatively clear boundary was 2.85 ± 0.21 mm. The presence of local tumor cell infiltration could be observed in all pathological sections. This might be one of reasons why the low signal boundary of GCTB on MRI was not clear and smooth. All cases of GCTB with blurred boundaries had local tumor cell infiltration. The average depth was 4.83 ± 0.12 mm. This indicated that the infiltration of local tumor cells always existed in the area where the GCTB appeared as a blurred boundary on MRI. The local tumor cell infiltration depth with blurred boundaries on MRI was the largest. From the point of view of medical imaging diagnosis, a clear and smooth boundary often means that there is no tumor cell infiltration in the region. There is often tumor cell infiltration in regions with relatively clear and blurred boundaries. However, histopathological studies show that most cases have different degrees of tumor cell infiltration regardless of clear, relatively clear, and blurred boundaries on MRI.

Previously, we reported that the peripheral tissue of GCTB consisted of GCTSC with high MMP‐9 staining degree and mRNA expression. This indicated that GCTB tumor cells located in peripheral tissue had stronger infiltration ability than in surrounding tissue^17^. This might be one of the reasons for local infiltration in many cases of GCTB.

In conclusion, the boundary displayed on MRI does not fully represent the intralesional curettage boundary. The intralesional curettage boundary where shown as clear, relatively clear, and blurred on MRI needs to be increased by at least 2 mm, 4 mm, and 6 mm, respectively.

We suggest that radiologists should show the GCTB boundary more accurately and pay more attention to the characteristics of the boundary to provide more accurate data of the intralesional curettage boundary. This might be an effective method to minimize residual tumor cells and reduce the postoperative recurrence rate.

Several limitations should be mentioned. First, only a small number of patients were evaluated, which limited the substantiality of the statistical results. More patients and multi‐institutional studies are needed to confirm our results. Second, CT images and 3D reconstructed images can also show GCTB boundaries clearly. However, due to more accurate diagnosis of GCTB by MRI, some patients did not have CT examination. We will add CT examinations in future studies. Third, although HE staining can show the depth of local tumor cell infiltration clearly, we still need other methods, such as immunohistochemistry, for further verification.

This is a preliminary study of the combination of MRI, pathology, and clinical surgery for more accurate assessment of the GCTB boundary. This study provides valuable information for intralesional curettage and precise surgical treatment using artificial intelligence in the future.
